# YOLOv8-segANDcal: segmentation, extraction, and calculation of soybean radicle features

**DOI:** 10.3389/fpls.2024.1425100

**Published:** 2024-07-11

**Authors:** Yijie Wu, Zhengjun Li, Haoyu Jiang, Qianyun Li, Jinxin Qiao, Feng Pan, Xiuqing Fu, Biao Guo

**Affiliations:** ^1^ College of Artificial Intelligence, Nanjing Agricultural University, Nanjing, China; ^2^ College of Engineering, Nanjing Agricultural University, Nanjing, China; ^3^ Cotton Research Institute, Xinjiang Academy of Agricultural and Reclamation Sciences, Shihezi, China; ^4^ Institute of Mechanical Equipment, Xinjiang Academy of Agricultural and Reclamation Science, Shihezi, China

**Keywords:** YOLOv8, soybean germination, image segmentation, feature extraction, radicle length calculation

## Abstract

The high-throughput and full-time acquisition of images of crop growth processes, and the analysis of the morphological parameters of their features, is the foundation for achieving fast breeding technology, thereby accelerating the exploration of germplasm resources and variety selection by crop breeders. The evolution of embryonic soybean radicle characteristics during germination is an important indicator of soybean seed vitality, which directly affects the subsequent growth process and yield of soybeans. In order to address the time-consuming and labor-intensive manual measurement of embryonic radicle characteristics, as well as the issue of large errors, this paper utilizes continuous time-series crop growth vitality monitoring system to collect full-time sequence images of soybean germination. By introducing the attention mechanism SegNext_Attention, improving the Segment module, and adding the CAL module, a YOLOv8-segANDcal model for the segmentation and extraction of soybean embryonic radicle features and radicle length calculation was constructed. Compared to the YOLOv8-seg model, the model respectively improved the detection and segmentation of embryonic radicles by 2% and 1% in mAP_50-95_, and calculated the contour features and radicle length of the embryonic radicles, obtaining the morphological evolution of the embryonic radicle contour features over germination time. This model provides a rapid and accurate method for crop breeders and agronomists to select crop varieties.

## 1 Introduction

Soybean (*Glycine max* (L.) Merr.) is an important annual leguminous plant (Fehr and Hadley, 1980). Soybean is a significant oilseed crop. From 2003 to 2022, the cumulative import volume of soybeans in China accounted for as high as 94.4% of the total oilseed imports (Zhang and Li, 2023). In order to ensure the security of oilseed and grain supplies and improve agricultural planting structures (Abbate et al., 2004), China has expanded soybean cultivation. Rapid breeding of soybeans in different geographical environments is crucial for increasing soybean yields (Turner et al., 2001). Assessing the vitality of soybean seeds through the evolution of embryonic radicle characteristics during germination is an important method for soybean selection and breeding (Kanjoo et al., 2012). Traditional research on embryonic radicle characteristics has been implemented by planting personnel through planting experience, manual calculations, and weighing, which is time-consuming, labor-intensive, subjectively judged, and lacks accuracy (Grinblat et al., 2016; Ahmed et al., 2019). Therefore, there is an urgent need to propose a rapid, accurate, and automated method for the segmentation, extraction, and calculation of soybean seed embryonic radicle characteristics.

As an important application of deep learning, the high accuracy and high throughput of machine vision recognition play a significant role in the agricultural field (Davies, 2009). The developed target detection model can determine the type and location of the target, especially the YOLO (you only look once) algorithm (Redmon et al., 2016), which stands out among a series of visual models due to its speed, accuracy, convenience, excellent detection, tracking, and segmentation functions, serving as a central target detection model in robotics, unmanned driving, and video surveillance (Terven et al., 2023). In the agricultural field, scholars worldwide have specifically optimized the YOLO model to address complex problems in agricultural production processes. For example, Thakuria and Erkinbaev (2023) achieved automated real-time grading of rapeseed health by improving the network architecture of the YOLO model, and Li et al. (2023) built a tomato ripeness grading and counting model named MHSA-YOLOv8, obtaining grading accuracy of 86.4% and counting accuracy of 91.6%. Chen et al. (2024) based on the YOLOv8-seg model, constructed the YOLOv8-CML model for the segmentation recognition of melon ripening in smart agriculture. Sampurno et al. (2024) deployed the YOLOv8n-seg model on a robot weeding machine by calling the instance segmentation function of YOLOv8, achieving 76.70% segmentation accuracy for weeding. Wang et al. (2023a) applied the YOLOv8-seg model in a lychee picking robot system to extract the regions of interest (ROI) of lychee fruits and branches, facilitating the smooth completion of lychee fruit picking tasks by the machine. Yue et al. (2023) used SimConv to replace the traditional convolution in the YOLOv8-seg network and segmented healthy and diseased tomato plants in the growth stage, achieving higher accuracy with the improved model.

The goal of object detection technology is to identify the position and category of specific objects in images or videos, usually outputting the bounding boxes and category labels of the objects. Image segmentation, as an extension of object detection, not only identifies the position and category of objects, but also provides detailed pixel-level object instances. This refined information is of great significance in many application areas, especially in agricultural production (Bai et al., 2023). However, the scope of these studies has been limited to improving the accuracy of model detection and segmentation of target objects, as well as increasing the lightweight nature of the model. YOLOv8 model, is an open-source YOLO model developed by Ultralytics based on YOLOv5, which features object detection, classification, pose recognition, and image segmentation (Terven et al., 2023). YOLOv8 is a cutting-edge, state-of-the-art model. It introduces multi-scale prediction techniques and utilizes more advanced feature extraction networks, enabling better detection of objects of varying sizes and extracting richer, more distinctive features from images. In contrast to other YOLO models such as YOLOv1, YOLOv2, and YOLOv3, which do not have image segmentation capabilities, they were not considered in the model selection process. The YOLOv5 model, with 6.9G FLOPS size, sacrifices segmentation recognition accuracy for lightweight deployment, resulting in poor segmentation accuracy for soybean root analysis and inadequate recognition accuracy for subsequent analysis. While YOLOv7 achieves high segmentation accuracy, its complex network structure with 141.9G FLOPS size makes it unsuitable for deployment on low-computing power agricultural production inspection devices. Considering the lightweight nature of the model and segmentation accuracy, we have chosen the YOLOv8 model, which combines the advantages of lightweight deployment and high segmentation recognition accuracy. The YOLO model architecture implements instance segmentation by utilizing the largest feature map size as the input for the Mask branch. Through convolutional layers, mask features are extracted. In comparison to other instance segmentation model frameworks such as Mask R-CNN and DeepLab, the YOLO model framework integrates object detection and instance segmentation functionalities into a unified framework, simplifying the processes of target recognition and pixel segmentation. Additionally, the YOLO series algorithms are designed to be simpler and more lightweight, facilitating deployment on edge devices. The open network structure and abundant open-source resources of the YOLO model enable researchers to easily improve and upgrade the model based on the YOLO network structure.

Previous studies have typically used the YOLO model to detect, recognize, and segment the desired targets. However, for the study of soybean embryonic characteristics, merely completing the detection and segmentation tasks is insufficient to determine the viability of soybean seeds. Furthermore, the embryonic characteristics of soybean seeds are complex, with issues such as internal bending and intertwining of radicle and shoot during seed embryonic growth (Scheres et al., 1995), making it more difficult to extract soybean embryonic characteristics and increasing the difficulty of seed identification for the model. Additionally, the YOLO model has poor accuracy in identifying small feature objects, so improving the model and increasing its accuracy is essential. Furthermore, if only the detection and segmentation of soybean radicles are conducted, it is only possible to determine the germination rate of soybeans, and the embryonic characteristics are still difficult to determine. Therefore, it is necessary to improve the output results of the YOLO model segmentation and to add a module for post-processing of the output images in the model. In response to these issues, this paper proposes a model called YOLOv8-segANDCal, specifically for the extraction of soybean seed germination embryonic characteristics and radicle length calculation. The following work will be conducted:

(1) Add a new convolutional attention framework, SegNext_Attention, to the YOLOv8-seg model to improve the accuracy of model detection and segmentation, specifically targeting the intricate and complex characteristics of soybean radicles.
(2) Modify the function for calling the prediction and segmentation function of YOLOv8-seg to output a binary mask image along with the segmented image, facilitating the subsequent extraction and calculation of soybean embryonic characteristics using the binary mask image.
(3) Add a calculation (Cal) module to the YOLOv8 network to compute the contour length of soybean radicles, which is used to accurately assess the growth status of soybean seed radicles.
(4) Provide a method for converting the contour length calculated by the Cal module into the actual length of soybean radicles.
(5) Utilize the model and a high-throughput, all-time monitoring system to obtain the morphological evolution of embryonic contour characteristics during germination.

The following chapters will begin by introducing the experimental equipment and methods, including the continuous time-series crop growth vitality monitoring system used and the data collection and preprocessing techniques. Subsequently, a detailed description of the soybean radicle segmentation design based on YOLOv8-seg will be provided. The paper will then present model evaluation metrics. In the Results and Discussion section, the paper will showcase model training results, comparative experimental results with other models, as well as ablation experiments and the performance of soybean radicle length conversion calculations. Finally, the paper will summarize the research findings, discuss limitations, and outline future research directions.

## 2 Materials and methods

### 2.1 Experiment equipment

**Figure 1:**
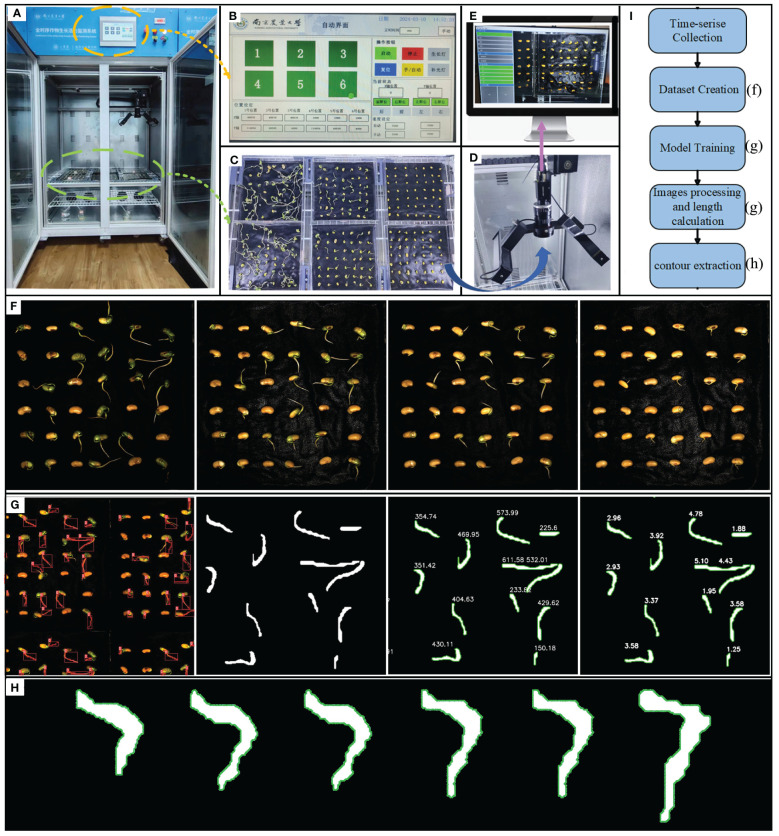
**(A)** Continuous time-series crop growth vitality monitoring system. **(B)** automatic control interface, which can configure the system. **(C)** seeds arranged in acrylic plates for upcoming photography, organized in a 3x2 layout **(D)** HIV VISION, camera for photography. **(E)** complimentary software. **(F)** image of the soybean germination. **(G)** model training, images processing and length calculation. **(H)** contour extraction. **(I)** experimental flow chart.

The continuous time-series crop growth vitality monitoring system we built (located in the Comprehensive Training Center of Mechanical Engineering at Nanjing Agricultural University, Nanjing) consists of an environmental control module for cultivation chambers and a rail image acquisition module, as depicted in **Figure 1**, with detailed system parameters outlined in **Table 1**. The germination chamber comprises a cultivation box measuring 1055mm in length, 740mm in width, and 1740mm in height (manufacturer: Henan Greentech Electric Technology Co., Ltd.). Positioned on the top side is a touchscreen, on the upper right side integrates power and LED switches, temperature adjustment buttons, and the interior of the box is divided into two layers by perforated partitions. The upper layer accommodates six acrylic cultivation trays and the rail image acquisition module, while the lower layer holds deionized water for experimental use. The box’s side features an embedded PTC hot air circulation system and a Tp-100 thermocouple to monitor the chamber’s temperature. The hot air circulation system operates to raise the temperature when it falls below the preset level and stops when it exceeds the upper limit to maintain temperature stability. Additionally, LED light sources are installed on the side of the box to supplement lighting. The HIV VISION RGB industrial camera, model MV-CS200–10GC, is equipped with a CMOS sensor, and a 30mm focal length telecentric lens (model LD-23–0.18X145, Supplier: Suzhou Youxin Zeda Co.). The camera is mounted on a stepper motor guide rail, allowing free movement within the upper plane of the box to capture images at six designated positions. Two LED light strips are installed below the camera to provide illumination during image capture, with an illuminance of 183.9K lux and brightness of 46.341 K cd/m^2^. The camera automatically triggers during the capture process and connects via a Gigabit Ethernet interface to transfer image data to a computer through the RJ-45 Ethernet interface at the back of the box, facilitating image editing, dataset construction, and object detection model training. This process ultimately enables the segmentation and extraction of soybean radicle characteristics and root length calculation. The specific steps are illustrated in **Figure 1**.

**Table 1:** Parameter settings for the system and its accompanying software.

Parameters	setup
Contant temperature	25°C
Distance from the camera to seeds	42.1cm
image saving format	.jpg
image saving resolution	2592×2048
image cropping format	.jpg
image cropping resolution	1500×1500
Shooting interval	20 minutes
Shooting duration	4 days

### 2.2 Data acquisition and pre-processing

#### 2.2.1 Data acquisition

**Figure 2:**
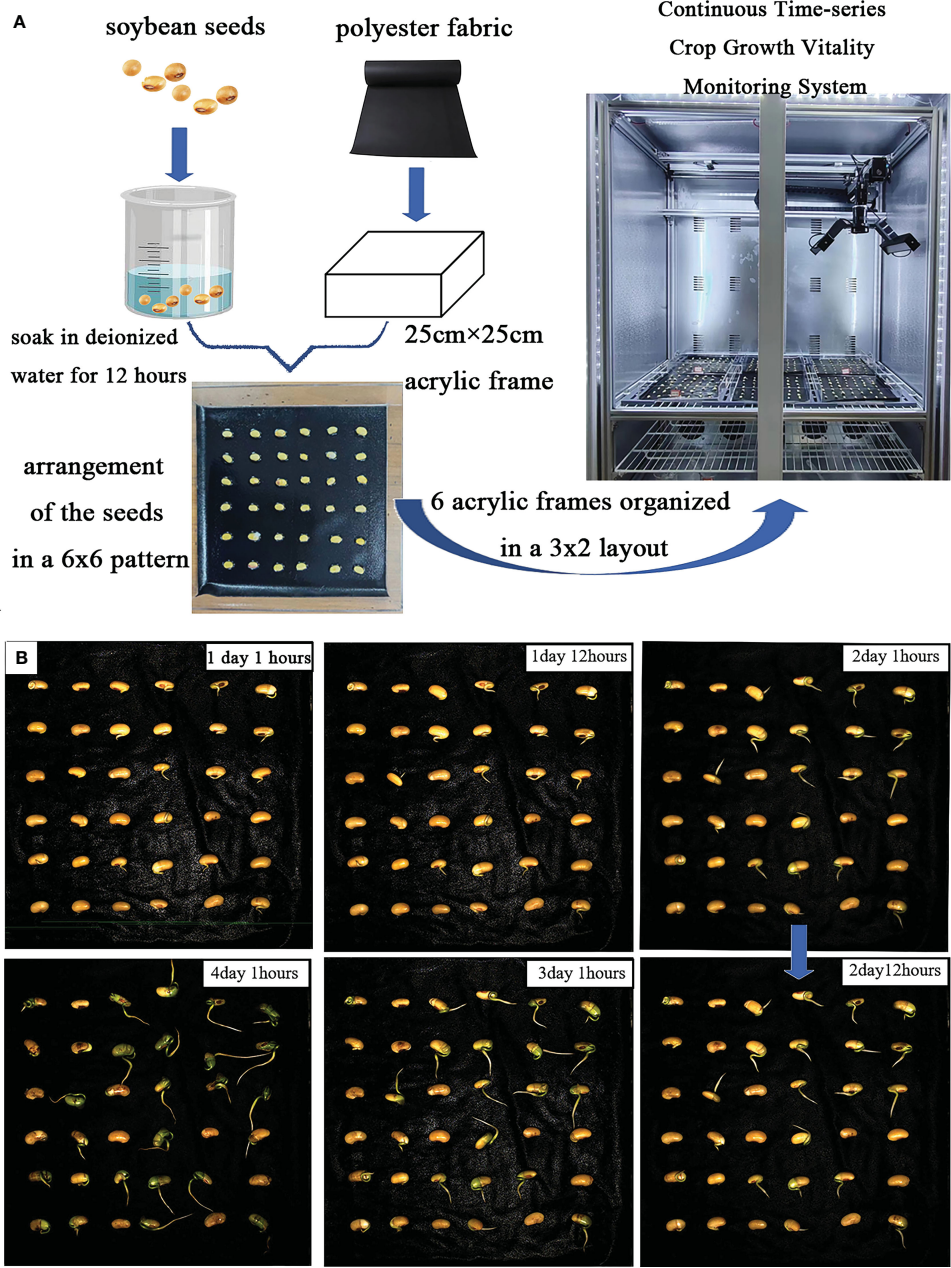
**(A)** demonstrates the experimental steps. **(B)** schematic of soybean seed growth process.

The experiment involved germinating soybean seeds, as depicted in **Figure 2A**. After soaking, the soybean seeds were arranged in a 6×6 layout on an acrylic frame for image acquisition. Throughout the experiment, deionized water was regularly sprayed. The germination characteristics of the soybean seed radicles are illustrated in **Figure 2B**, with noticeable differences in root development observed in images captured every 12 hours. Relevant experimental data is presented in **Table 2**.

**Table 2:** Experiment-related data.

data	setup
Seed varieties	Zhong huang 13
Number of seeds	216
Acrylic frame specifications	25cm×25cm
Number of selected images	600
image cropping resolution	1500×1500
Actual size of the image	25cm×25cm
The ratio of training, prediction and validation	6:2:2

#### 2.2.2 Data pre-processing

**Figure 3:**
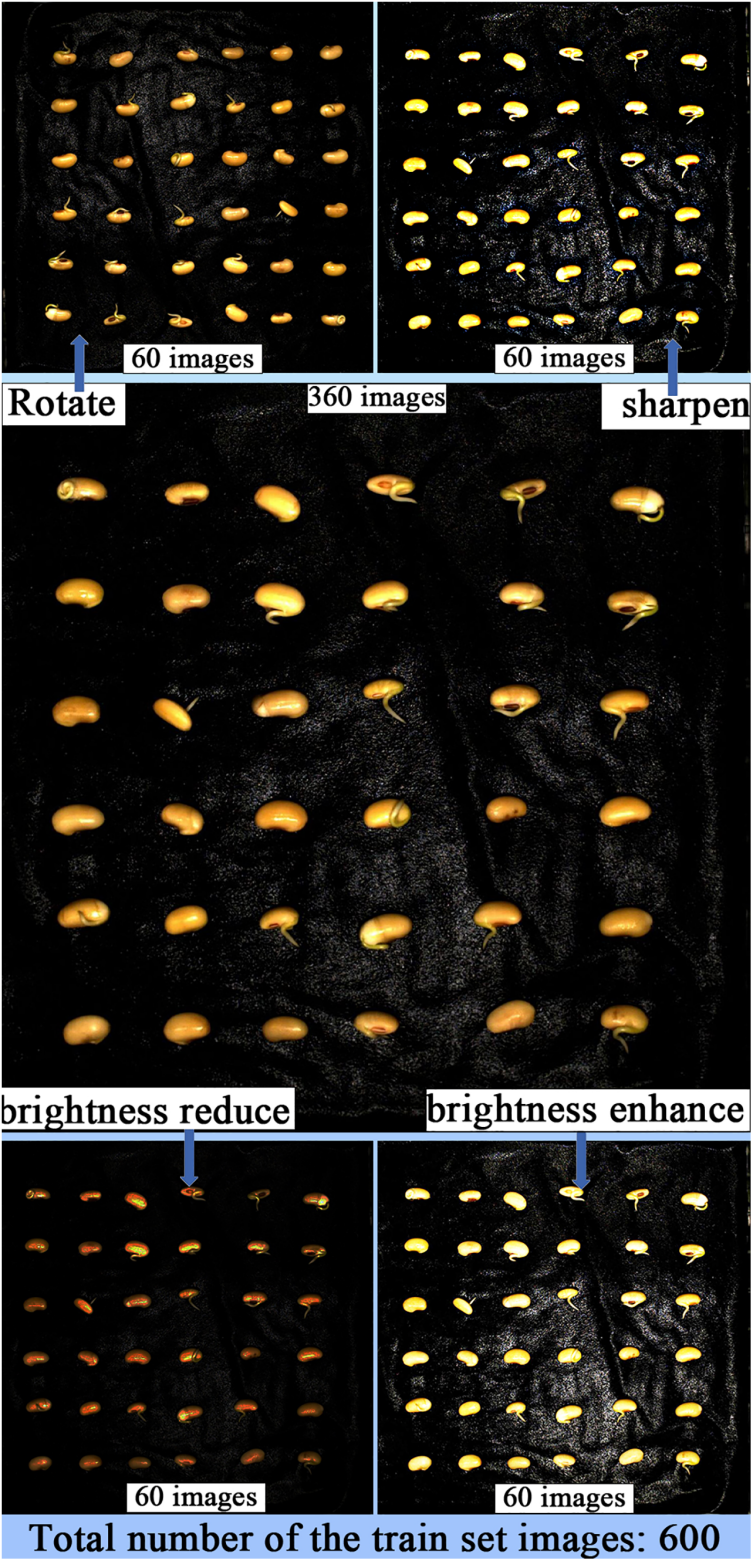
Augmenting the dataset through sharpening, rotation, and adjusting brightness.

In image segmentation tasks, data augmentation can help the model better recognize and segment target objects in images. It can improve the model’s generalization ability, reduce overfitting, enhance the model’s robustness, and improve the performance of classification, detection, and segmentation. When constructing the soybean radicle germination dataset, labeling the radicle data is time-consuming and costly. By using data augmentation, we can expand the dataset without increasing data collection costs. Since the radicle images are captured in our germination chamber, the lighting conditions are controlled by LEDs, with a constant illumination of 183.9 K lux and brightness of 46,341K cd/m^2^. To adapt the trained model to changes in environmental lighting during actual agricultural breeding processes, we applied brightness reduction and brightness enhancement data augmentation strategies to the soybean germination dataset. This was done to improve the model’s ability to extract features from soybean radicle images with different brightness levels. Furthermore, in the process of image collection in agricultural production, it is difficult to avoid overexposure when capturing images with cameras. By using the sharpening data augmentation strategy to simulate overexposed images, the model can better handle these seedling images with locally overexposed areas. During the soybean seed germination process, the seedling roots may twist and rotate, appearing at different angles. By employing the image rotation data augmentation strategy, the model can learn the features of the roots in different directions, enhancing the model’s ability to recognize and segment posture changes. If the model is trained only on non-rotated images, it may become overly sensitive to specific root features in certain directions. Rotation augmentation can reduce errors in classification caused by directional biases in the model. These data augmentation strategies expanded the training set to 600 images, with no data augmentation applied to the test and validation sets. **Figure 3** illustrates the images after data augmentation.

**Figure 4:**
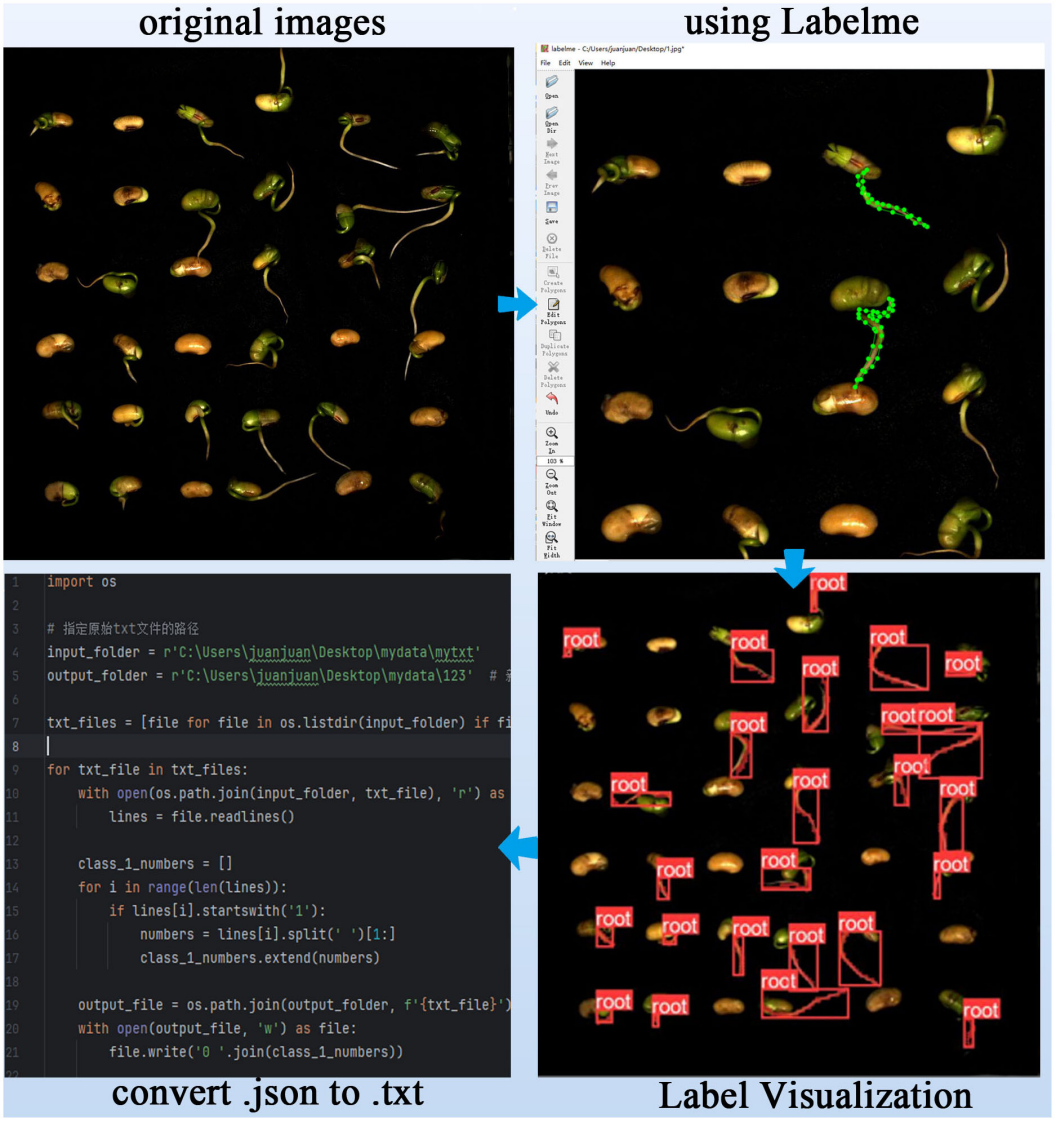
Dataset processing workflow.

We manually performed semantic segmentation labeling of the positions of soybean seed radicles using the annotation software LabelMe (version 3.16.7, manufacturer: MIT Computer Science and Artificial Intelligence Laboratory (CSAIL).) to create a complete training dataset. **Figure 4** illustrates the pixel-level labeling process for soybean seed radicles. As shown in **Figure 4**, the process involved importing the dataset folder into the LabelMe software, using polygon annotations to mark the radicle area, and saving the annotations as a.json file. Finally, a program script was used to convert the.json file to a.txt file for model training.

### 2.3 YOLOv8-segANDcal soybean radicle segmentation design base on YOLOV8-seg

The structure of the YOLOv8-seg model is consisted of two modules: the Backbone and the Head. The “conv” convolution is used to progressively extract image features (Jocher et al., 2020). The C2f module is designed with reference to the C3 module and the concept of ELAN, enabling YOLOv8 to obtain richer gradient flow information while ensuring lightweight characteristics (Wang et al., 2023b). The SPPF (Spatial Pyramid Pooling-Fast) module is adapted to handle objects of different sizes, allowing the model to extract features across multiple dimensions and increasing its adaptability to detect object sizes (Jiang et al., 2023).

**Figure 5:**
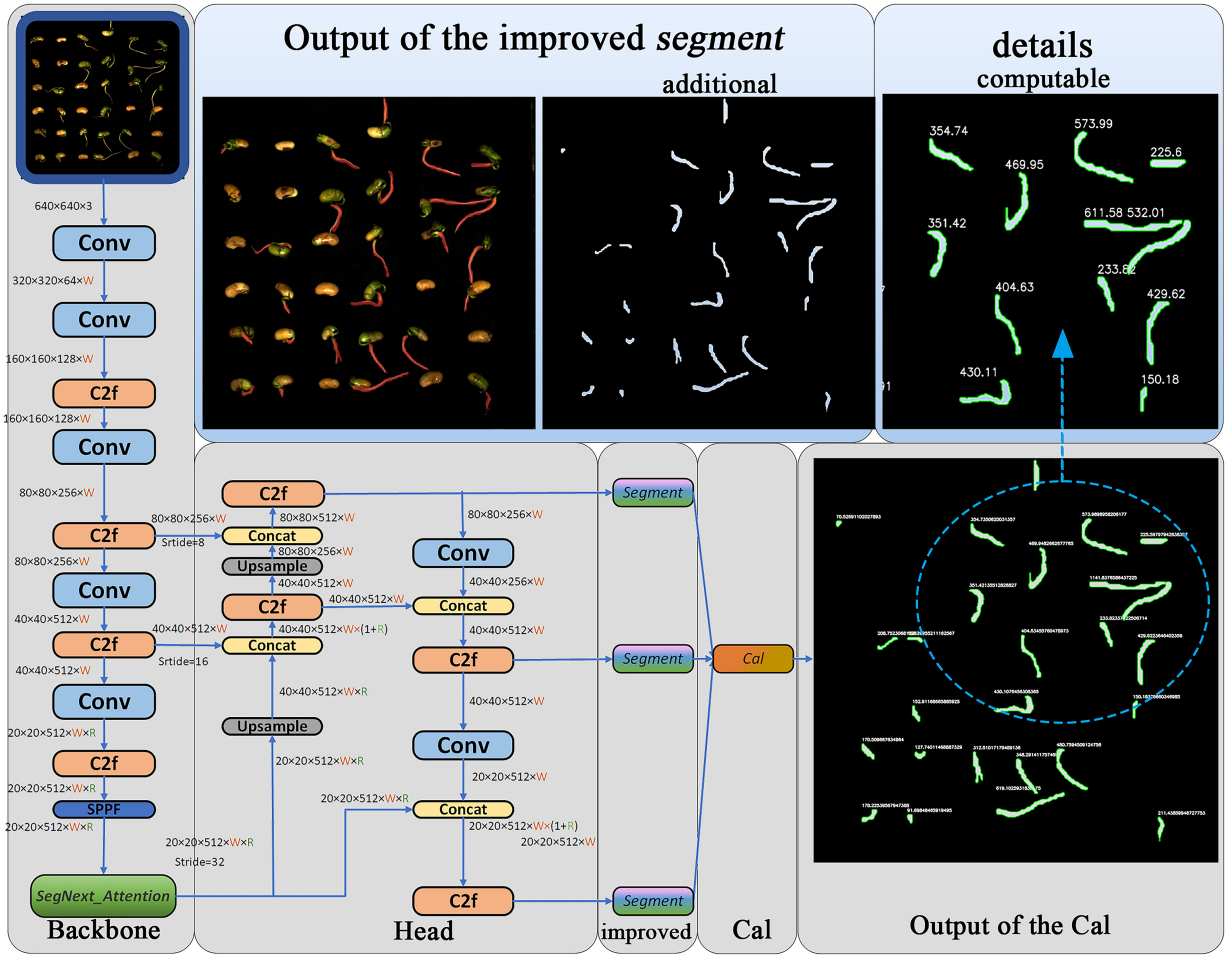
Visualization of YOLOv8-segANDcal structure.

In order to improve the accuracy of soybean seed radicle detection, enhance the practicality of segmented image outputs during the prediction phase, and equip the model with the ability to compute soybean seed radicle phenotypes, the following improvements were made:1)added the SegNext_Attention attention framework structure after the SPPF module located in the Backbone position. This helps the model better utilize global contextual information, enabling the model to more accurately focus on the regions of interest while ignoring background areas, thus improving the model’s detection accuracy.2)modified the Segment module of the YOLOv8-seg at the prediction stage. When the model predicts and generates images containing image segmentation information, it directly draws the corresponding binary mask images by reading the ‘masks’ values of the models generated “results”, enhancing the practicality of the model’s output images.3)added a calculation (Cal) module at the end of the two-stage Backbone and head modules of YOLOv8. This module utilizes the Canny algorithm composed of Gaussian filtering, non-maximum suppression, gradient calculation, and double threshold detection to calculate the edge length of the mask binary image, thereby determining the root length of the soybean seed obtained through calculation, and assessing the soybean seed radicle characteristics. The Cal module equips the YOLOv8-segANDcal model with more comprehensive functionality for computing soybean radicle features. **Figure 5** illustrates the structure of the YOLOv8-segANDcal model. The following sections will sequentially introduce the design and implementation of the SegNext_Attention module, the improved Segment module, and the Cal module.

#### 2.3.1 SegNext_Attention

**Figure 6:**
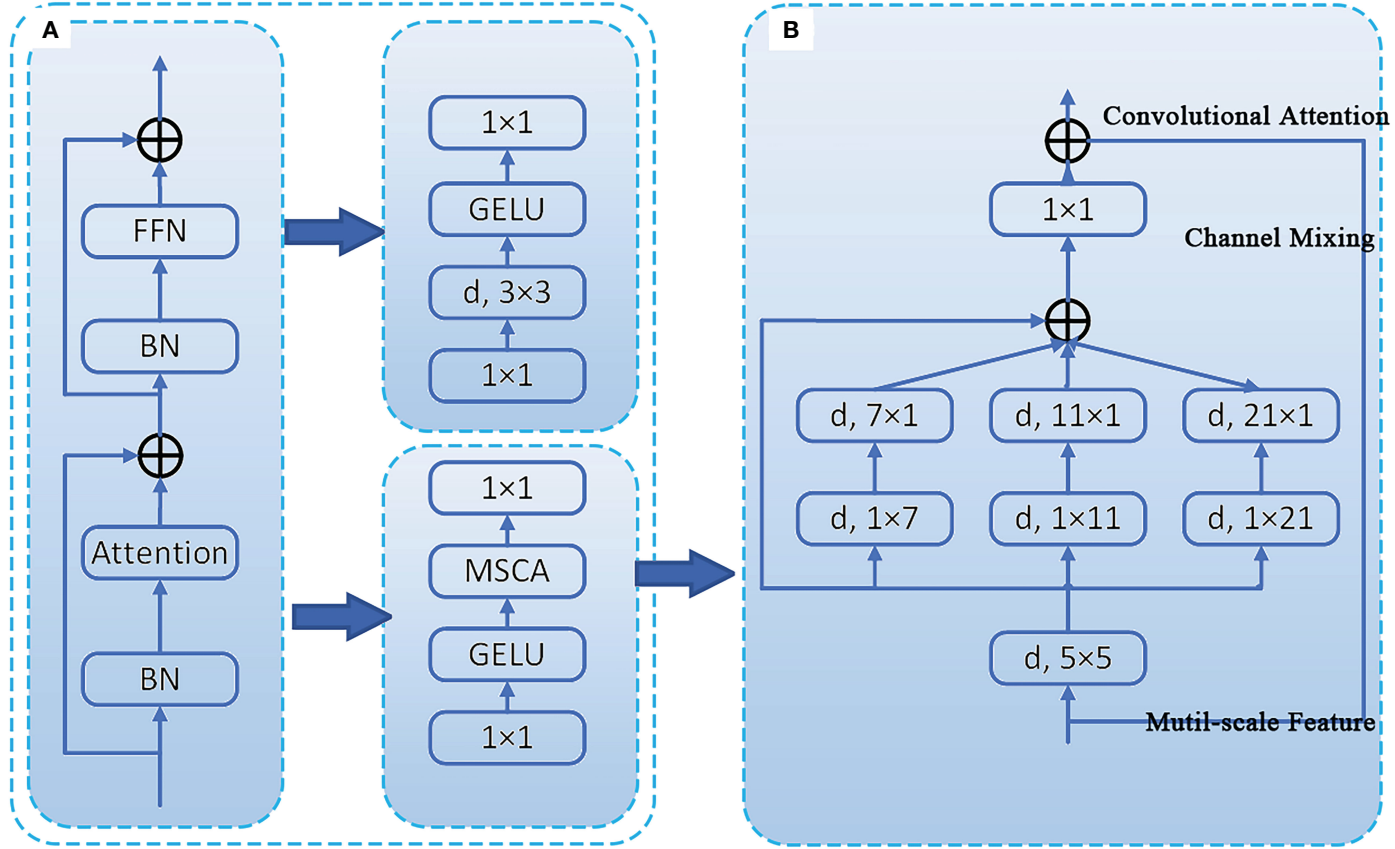
Illustration of the proposed MSCA and MSCAN. **(A)** the stage of MSCAN. **(B)** the structure of MSCA.

SegNext_Attention is a simple convolutional network architecture designed for semantic segmentation, consisting of an encoder, attention mechanism, decoder, and loss function. It uses a strong backbone network as the encoder, which enables multi-scale information interaction and effectively addresses the problem of large-scale differences in semantic segmentation targets (Guo et al., 2022). The encoder (MSCAN) structure of this module is shown in **Figure 6A**. The novel multi-scale convolutional attention (MSCA) module within it comprises three parts: deep convolution for aggregating local information, multi-branch deep stripe convolution for capturing multi-scale context, and 1×1 convolution for modeling relationships between different channels. The structure of the MSCA module is illustrated in **Figure 6B**.

The input of MSCA is the output of a 1×1 convolution. The MSCA module can be expressed in the following mathematical form (1, 2), where ***F*** represents the input feature, and $Scale_{i}$ , ***i∈{0,1,2,3}***, represents the branches in **Figure 2B**. Branch 0 denotes a skip connection, while the other branches use depth-wise separable convolutions to approximate large convolutional kernels (approximating 7x7, 11x11, and 21x21, respectively).


(1) $$Att=Conv_{1\times 1}\left(\sum_{i=0}^{3}Scale_{i}(DW-Conv(F))\right)$$

(2) Out=Att ⊗F

MSCAN consists of 4 stages with reduced spatial resolutions, each stage containing a downsampling block and a stack of MSCA. The downsampling block is implemented by a 3×3 convolution with a stride of 2, followed by batch normalization (Ioffe and Szegedy, 2015). Batch normalization improves segmentation performance. Integrating SegNext_Attention into the SPPF module located in the Backbone position increases the computational overhead of the system. However, it enhances the image segmentation accuracy of soybean radicle.

#### 2.3.2 Improved segment

**Figure 7:**
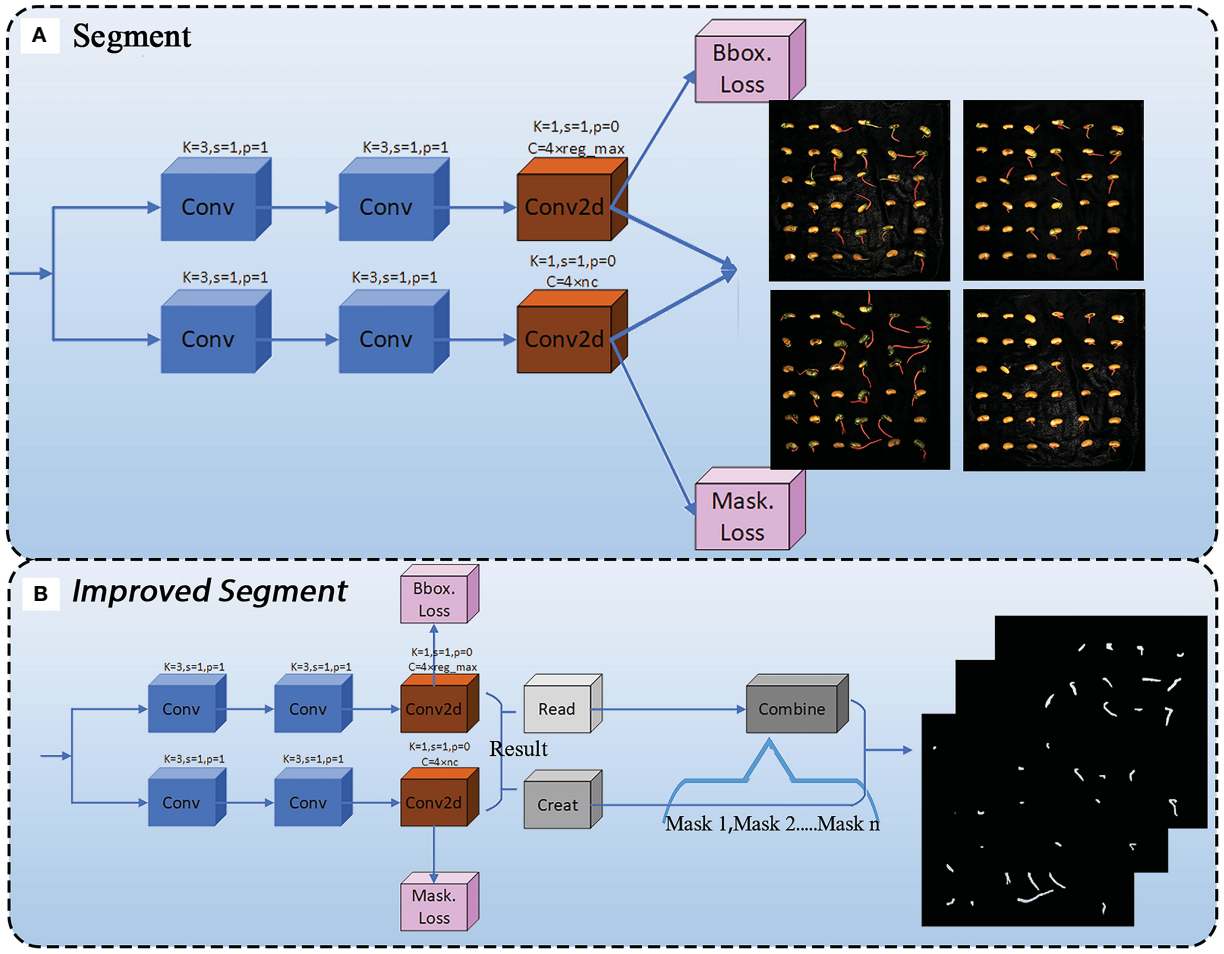
**(A)** Segment module before improvement. **(B)** Segment module after improvement.

For the extraction of soybean radicle features, we have improved the Segment module. During the prediction phase, when the entire model’s segmentation prediction function is called, the Segment detection head will output images with segmentation examples, as shown in **Figure 7A**. Due to the superimposition of pixel-level annotations on the original images and the relatively small size of the soybean radicle compared to the entire seed, the displayed results are not sufficiently clear. In order to facilitate the subsequent processing of the images and ensure the feasibility of extracting radicle contours, we have made improvements to the Segment module. The improved Segment module is shown in **Figure 7B**.

Before the improvement, the ‘‘Segment’’ module directly output the segmented image through two convolutions and a 2D convolution (conv2d). However, the resulting ‘‘result’’ object contains a large amount of unused information. According to the ultralytics official documentation, the available attribute objects are shown in **Table 3**.

**Table 3:** The types of attributes that can be used in the result and their descriptions.

Attribute	Type	Description
orig_img	numpy.ndarray	The original image as a numpy array.
orig_shape	tuple	The original image shape in (height, width) format
boxes	Boxes, optional	A Boxes object containing the detection bounding boxes
Masks	Masks, optional	A Masks object containing the detection masks
Probs	Probs, optional	A Probs object containing probabilities of each class for classification task
Keypoints	Keypoints, optional	A Keypoints object containing detected keypoints for each object
Speed	dict	A dictionary of preprocess,inference,and postprocess speeds in milliseconds per image
names	dict	A dictionary of class names
path	str	The path to the image file

This includes an attribute Masks, which is a Masks object containing the detection masks after segmentation. To fully utilize this object, we first used the Read module to read the mask data. At the same time, the Create module creates an empty mask of the same size as the original image, which is mainly used to store the results of merging multiple masks. Since a single image contains multiple germinated seeds, and therefore multiple masks after radicle segmentation, a results object contains multiple masks attributes. To address this, the Combine module iterates through each mask, converts it to type uint8, adjusts its size to match the original image, and then adds the masks together to obtain a new merged mask containing all the mask information. Finally, the merged mask is subjected to threshold processing to ensure that the values are within the range of [0, 255], and the merged mask image is output and saved in the form of a file named “in.png” with a resolution of 1500×1500. The improved Segment module enables the model to directly output binary images during the prediction phase, with the images containing only 0 and 1 data, greatly facilitating subsequent image processing.

#### 2.3.3 CAL

**Figure 8:**
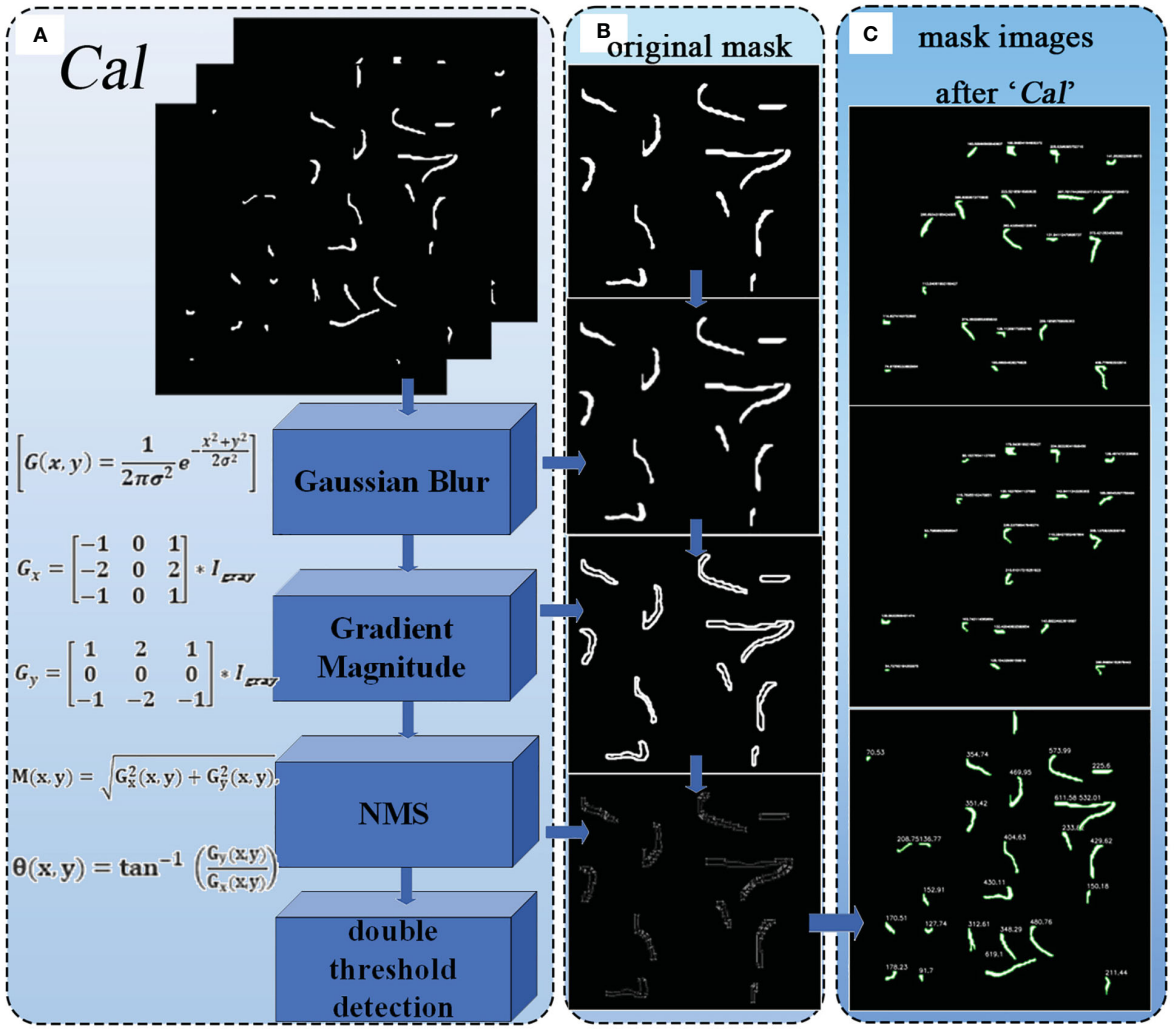
**(A)** Structure of the Cal module. **(B)** Various results of the image after being processed by the algorithm. **(C)** The output images of the "cal" module.

As mentioned above, the improved Segment module outputs a binary image containing merged mask information, obtaining the radicle feature map of germinated soybean seeds. To further obtain the specific radicle length of soybeans, we added the Cal module for calculation and measurement. Its structure is shown in **Figure 8A**, where the Canny edge detection algorithm (Ding and Goshtasby, 2001)is used to calculate the number of edge contour pixels in the binary image. It is composed of Gaussian filtering, gradient calculation, non-maximum suppression (NMS), and double threshold detection. After gradient calculation, the edge positions and directions in the image can be accurately determined (Sozer et al., 2014). Applying non-maximum suppression to the gradient image (Bodla et al., 2017) can yield finer edges. Using double thresholding (Fan et al., 2015), the gradient image can be divided into strong and weak edges, and the final edges are determined based on their connectivity. **Figure 8B** shows the results of the example image after processing with these algorithms. By cumulatively adding the number of edge contour pixels processed by the Canny algorithm, we ultimately obtain the length of the edges (in terms of pixel count). The contours are outlined, and the length (in terms of pixel count) is displayed next to each contour, as shown in **Figure 8C**.

In the entire YOLOv8-segANDcal model, the Cal module operates after the prediction phase, thus being independent of the original Backbone and head modules. The Cal module was not utilized during the training process of the model. Therefore, the addition of the Cal module has minimal impact on the overall lightweight nature of the model.

### 2.4 Evaluation metrics

#### 2.4.1 Evaluation metrics of soybean radicle

The radicle characteristics of soybean seeds directly reflect the germination and growth status of soybean seeds (Gregory, 2006). As mentioned earlier, through the Cal module, we have obtained the pixel count of the radicle feature boundary, and the size of the cropped image is 1500×1500 pixels, corresponding to an actual length of 25 cm×25 cm. Therefore, using Formulas (3) and (4) to convert and calculate the length of the soybean radicle, the length is half of the irregular polygon shape formed by the radicle after segmentation calculation (Wang et al., 2023c).


(3) R=actual_length/image_unilateral_pixel

(4) Radicle_length=sum_pixels/2×R

**Figure 9:**
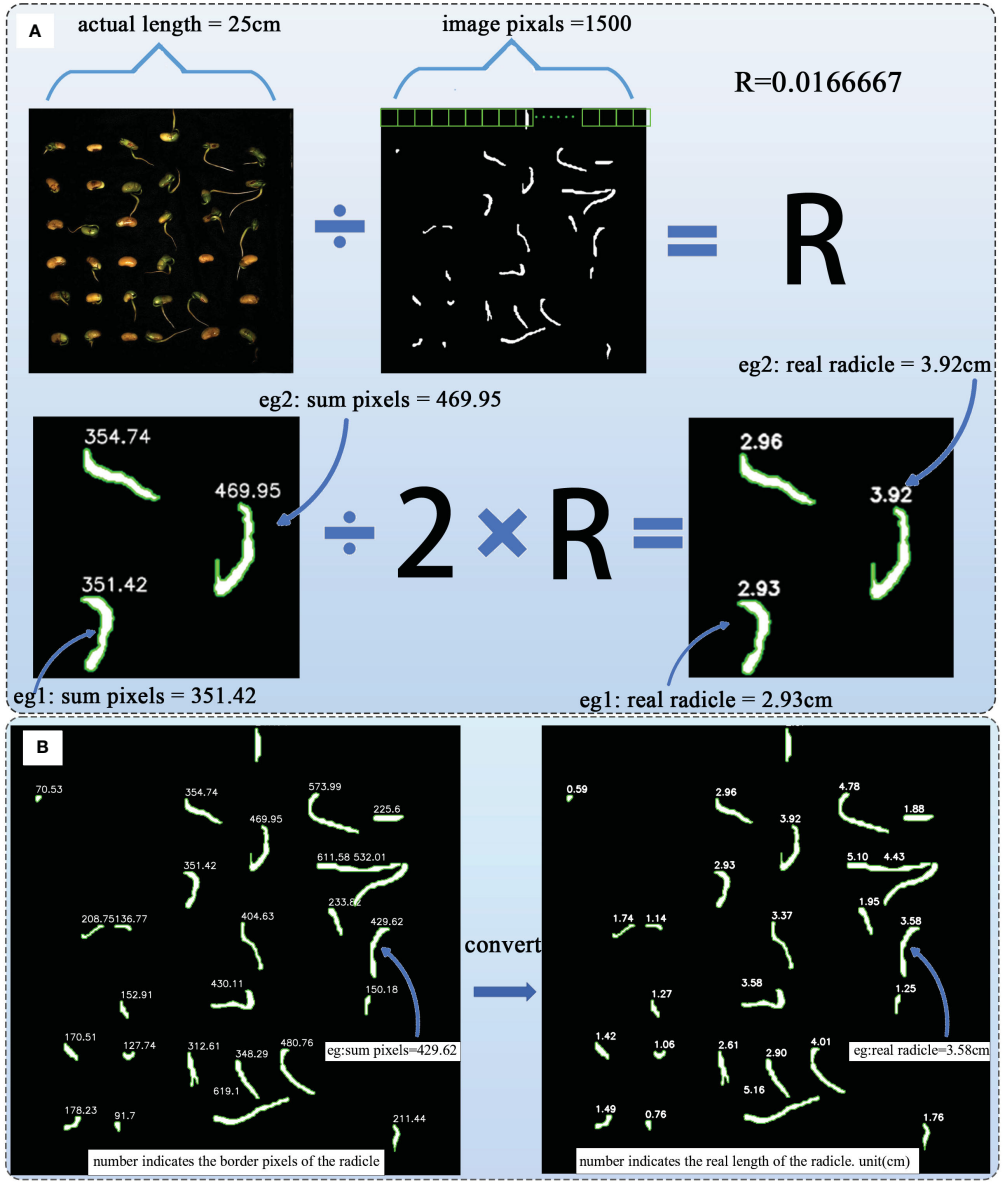
**(A)** The process of radicle length transformation calculation. **(B)** Example of actual radicle length after calculation.

The formula contains the constant ***actual_length***, which is 25cm, and the fixed constant ***image _unilateral _pixel***, which is 1500, representing the pixel value of one side of the image. ***R*** is the proportion calculated, ***sum_pixels*** is the number of border pixels obtained after the Cal module calculation. By substituting these parameters into Formula (4), the actual radicle length can be obtained. **Figure 9A** illustrates the process of this radicle length conversion calculation. **Figure 9B** shows an example of actual radicle length after calculation.

#### 2.4.2 Model evaluation metrics

The average precision (***mAP_0.5_*** and ***mAP_0.5-0.95_***) is used to evaluate the model’s detection and segmentation accuracy for soybeans. They can be represented using Formulas (5) and (6).


(5) $$mAP_{0.5}=\frac{1}{n_{c}}\int_{0}^{1}P(R)dR$$

(6) $$mAP_{0.5-0.95}=avg\left(mAP_{i}\right),i=0.5:0.05:0.95$$

The higher the average precision, the better the model’s actual performance in detecting and segmenting soybean radicles. Additionally, the model’s lightweight nature and complexity are evaluated using ***params*** and ***FLOPS***,which are given by Formulas (7) and (8). ***K^2^*** represents the size of the convolutional kernel, and ***H×W*** represents the height of the input feature map multiplied by the width of the input feature map. ***C_out_*** represents the number of output channels, and ***C_in_*** represents the number of input channels.


(7) $$FLOPS=2\times H\times W\left(C_{in}K^{2}+1\right)C_{\text{out }}$$

(8) $$\text{Params}=C_{\text{in }}\times K^{2}\times C_{\text{out }}$$

## 3 Results and discussion

### 3.1 Training environment and hyperparameter settings

The model training was conducted on a Windows 10 platform with hardware configuration including NVIDIA GeForce RTX3050 Laptop GPU and 11th Gen Intel(R) Core(TM) i5–11400H @ 2.70Ghz. The deep learning framework used was PyTorch 1.9.0(manufacturer: Facebook Artificial Intelligence Research (FAIR).)with Python 3.8.18(manufacturer: Python Software Foundation(PSF).), and the CUDA version was 11.1(manufacturer: NVIDIA). **Table 4** shows the hyperparameter settings used before training various models for comparative experiments.

**Table 4:** Settings of hyperparameters.

Parameters	setup
Epoch	100
Batch size	1
NMS IoU	0.65
Image Size	640×640
Initial Learning Rate	1×10^-2^
Final Learning Rate	1×10^-4^
Momentum	0.937
Weight-Decay	1×10^-4^

### 3.2 Comparison experiments

**Figure 10:**
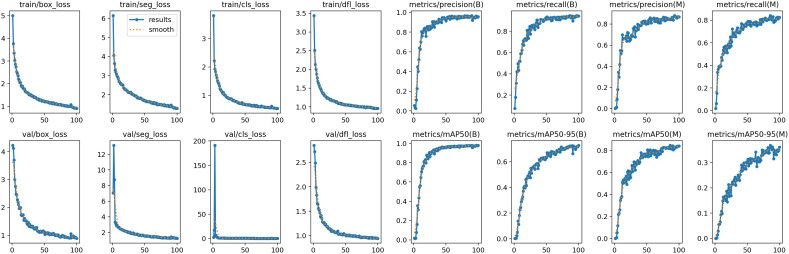
Model training plot of YOLOv8-segANDcal.

**Figure 10** shows the training plot of our model after training. To demonstrate the superior performance of our model in detecting and segmenting soybean radicle among similar models, we conducted comparative experiments with YOLOv5-seg, YOLOv7-seg, and YOLOv8-seg. **Table 5** presents the results obtained under the same configuration environment and hyperparameter settings. Despite our model having a larger number of FLOPS and parameters, it achieved the highest detection average precision and segmentation mask average precision. The detection mAP_50_ reached 97.7%, and mAP_50-95_ reached 72.2%. Furthermore, the segmentation mask mAP_50_ reached 84.6%, and mAP_50-95_ reached 36.8%, surpassing all other models. In comparison to the unimproved YOLOv8-seg, our model achieved a 2% improvement in detection mAP_50-95_ and a 1% improvement in segmentation mask mAP_50-95_. While YOLOv5 is the most lightweight model, it exhibited poor accuracy in segmenting soybean roots, with segmentation mAP_50_ and mAP_50-95_ reaching only 61.1% and 15.1%, respectively. Additionally, YOLOv8-segANDcal had only 1/5 of the GFLOPS of YOLOv7, yet it achieved a 0.3% higher detection mAP_50_ and a 9.7% higher segmentation mask mAP_50-95_.

**Table 5:** Experimental results of different models.

Model	Params (M)	FLOPs (G)	mAP^box^_50_ (%)	mAP^box^_50-95_ (%)	mAP^mask^_50_ (%)	mAP^mask^_50-95_ (%)
YOLOv5-seg	**1.8**	**6.9**	95.7	52.6	61.3	15.1
YOLOv7-seg	37.8	141.9	97.4	62.5	84.7	27.1
YOLOv8-seg	3.2	12	97.6	70.7	**85.0**	35.9
YOLOv8-segANDcal	6.5	27.7	**97.7**	**72.2**	84.6	**36.8**

Bold indicates the best experimental results.

The data from comparative experiments indicates that our model, while having a larger model size compared to the selected models, exhibits the best accuracy in detecting and segmenting soybean radicle features. Particularly, the average precision of the segmentation mask directly influences the accuracy of radicle length calculation. In order to facilitate the determination and calculation of soybean radicle length for subsequent selection analysis, ensuring a higher segmentation mask average precision is deemed acceptable, even if it results in additional computational overhead during the model training process.

### 3.3 Ablation experiments

To validate the effectiveness of the data augmentation strategies we employed, we conducted ablation experiments on the YOLOv8-segANDcal model. The specific results are presented in **Table 6**. It is evident that with the gradual addition of various data augmentation strategies, the model’s detection accuracy shows an overall increasing trend. After incorporating four data augmentation strategies, the model achieved a maximum mAP^box50-95^ of 72.2% and a maximum mAP^mask50-95^ of 36.8%. The data augmentation strategies expanded the dataset and improved the accuracy of the model for detection and segmentation.

**Table 6:** Data augmentation ablation experiment.

Data enhancement	Number of dataset	mAP^box^_50_ (%)	mAP^box^_50-95_ (%)	mAP^mask^_50_ (%)	mAP^mask^_50-95_ (%)
Original dataset	360	95.8	70.7	81.4	33.9
Original dataset+brightness reduce	420	96.3	70.6	82.3	34.1
Original dataset+brightness reduce+brightness enhance	480	96.7	71.3	83.7	34.4
Original dataset+brightness reduce+brightness enhance+rotate	540	**97.9**	71.9	83.6	36.1
Original dataset+brightness reduce+brightness enhance+rotate+sharpen	600	97.7	**72.2**	**84.6**	**36.8**

Bold indicates the best experimental results.

**Figure 11:**
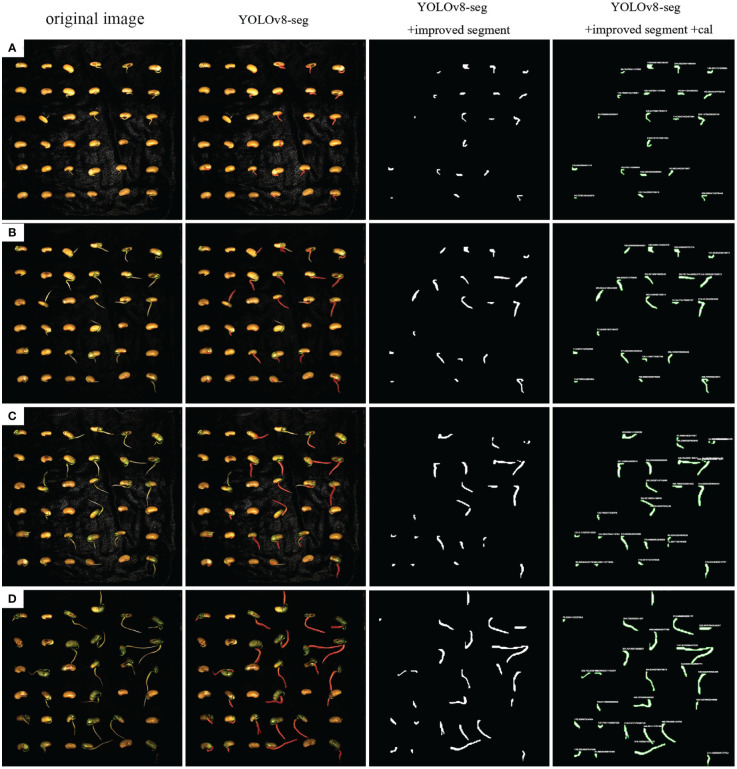
The performance of YOLOv8-segANDcal segmentation detection. **(A–D)** is the radicle length of seeds at different germination stages.

In order to specifically evaluate the effects of the improved Segment module and the added Cal module, we input four images of soybean seeds at different germination stages into the model. **Figures 11A-D** show soybean seeds with distinct radicle differences. It can be observed that the original YOLOv8 model output images with pixel-level masks, while the improved Segment module helped the model output corresponding binary images. With the addition of the Cal module, the model successfully provided the pixel length of the corresponding radicle contour boundaries, yielding comprehensive results.

### 3.4 Performance of soybean radicle length conversion calculation

**Figure 12:**
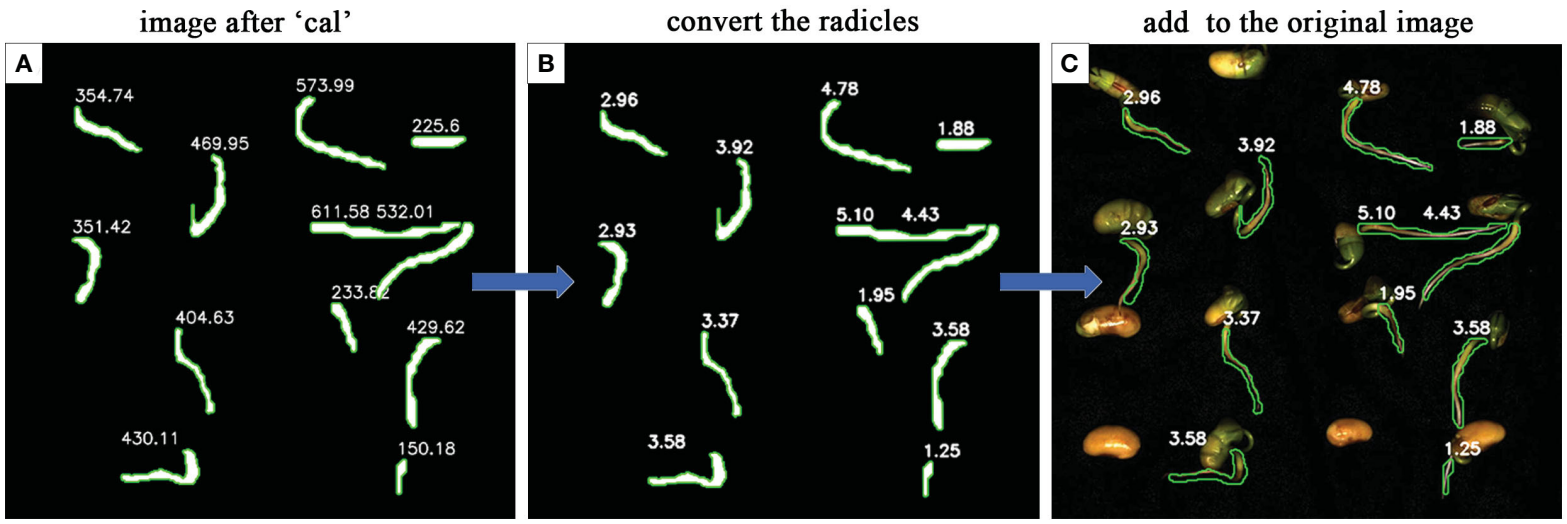
The performance of radicle length conversion methods. **(A)** is the mask image calculated after the 'Cal' module. **(B)** is the actual radicle length calculated using the radicle length conversion method. **(C)** the actual radicle length is superimposed on the original image.

To evaluate the effectiveness and accuracy of the radicle length conversion calculation, a subset of samples was selected, excluding seeds with segmentation errors from the model. Upon the calculation of the mask images by the ‘Cal’ module, the actual lengths of the radicles for each soybean seed were computed using the radicle length conversion method and superimposed onto the original images, showing in **Figure 12**.

**Figure 13:**
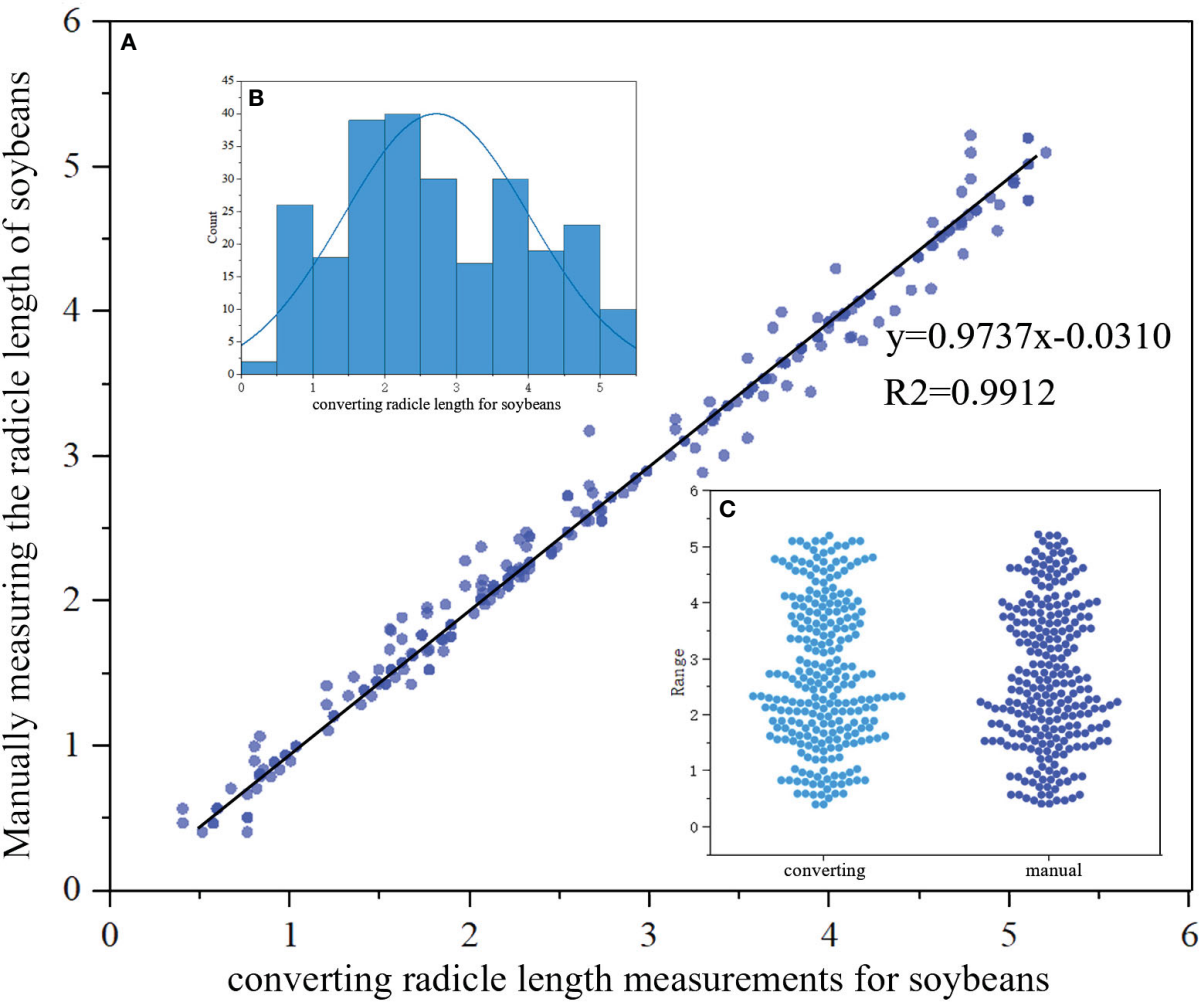
**(A)** Comparison between manual and converting radicle of the length of the soybean seed obtained in regression function images.Scatter plots represent discrete relationships, while lines represent linear relationships. **(B)** Distribution map of radicles. **(C)** Beeswarm plot of converting and manual.

We then conducted a regression analysis to compare the lengths obtained through the radicle length conversion method with the average radicle lengths obtained from manual measurements. The research results indicate that, for a selected quantity of samples, the lengths of germinated soybean radicles obtained through manual measurements ranged from 0.51 cm to 5.20 cm, with an average length of 2.66 cm and a median length of 2.47 cm. In contrast, the range of soybean radicle lengths obtained through machine vision and radicle length conversion calculation was 0.59 cm to 5.10 cm, with an average length of 2.71 cm and a median length of 2.50 cm. We employed linear regression analysis to compute the correlation coefficient between manual and machine vision measurements, with the corresponding results shown in **Figure 13A**. Additionally, the distribution of the calculated soybean radicle lengths is presented in **Figure 13B**, where it can be observed that the majority of radicle lengths are distributed around 2.5 cm. Furthermore, **Figure 13C** shows a beeswarm plot of the radicle lengths calculated by radicle length conversion and those manually marked, both exhibiting the highest density around 2.5 cm, with a relatively uniform distribution at other lengths.

The linear regression function between manual measurement and radicle length conversion measurement is y=0.9737x-0.0310, with a regression coefficient of 0.9737. Also, the goodness of fit value R2 is calculated to be 0.9912, which is close to 1, indicating a high degree of fit of the curve to the data. Hence, it can be considered that there is a high degree of fit and low error between manual measurement and radicle length conversion measurement techniques. In terms of measurement time, the manual measurement method consumes a significant amount of time and labor, while the YOLOv8-segANDcal model demonstrates faster speed in calculating soybean radicle lengths. The YOLOv8-segANDcal model not only efficiently and accurately segments soybean radicle images but also rapidly and precisely calculates soybean radicle lengths.

### 3.5 tracking of the full-time sequence of soybean radicle features

The continuous time-series crop growth vitality monitoring system we have built can continuously monitor subtle changes in soybean seed radicle features over a short period of time. Typical soybean seeds in the growing state were captured from continuous images labeled as 1, 2, 3, and 4. The time interval between the selected images was 1 hour. We utilized our model to segment and detect the radicles and, combined with the radicle length conversion method, calculated the actual radicle lengths during this time period to analyze the changes in soybean radicles over a 7-hour period.

**Figure 14:**
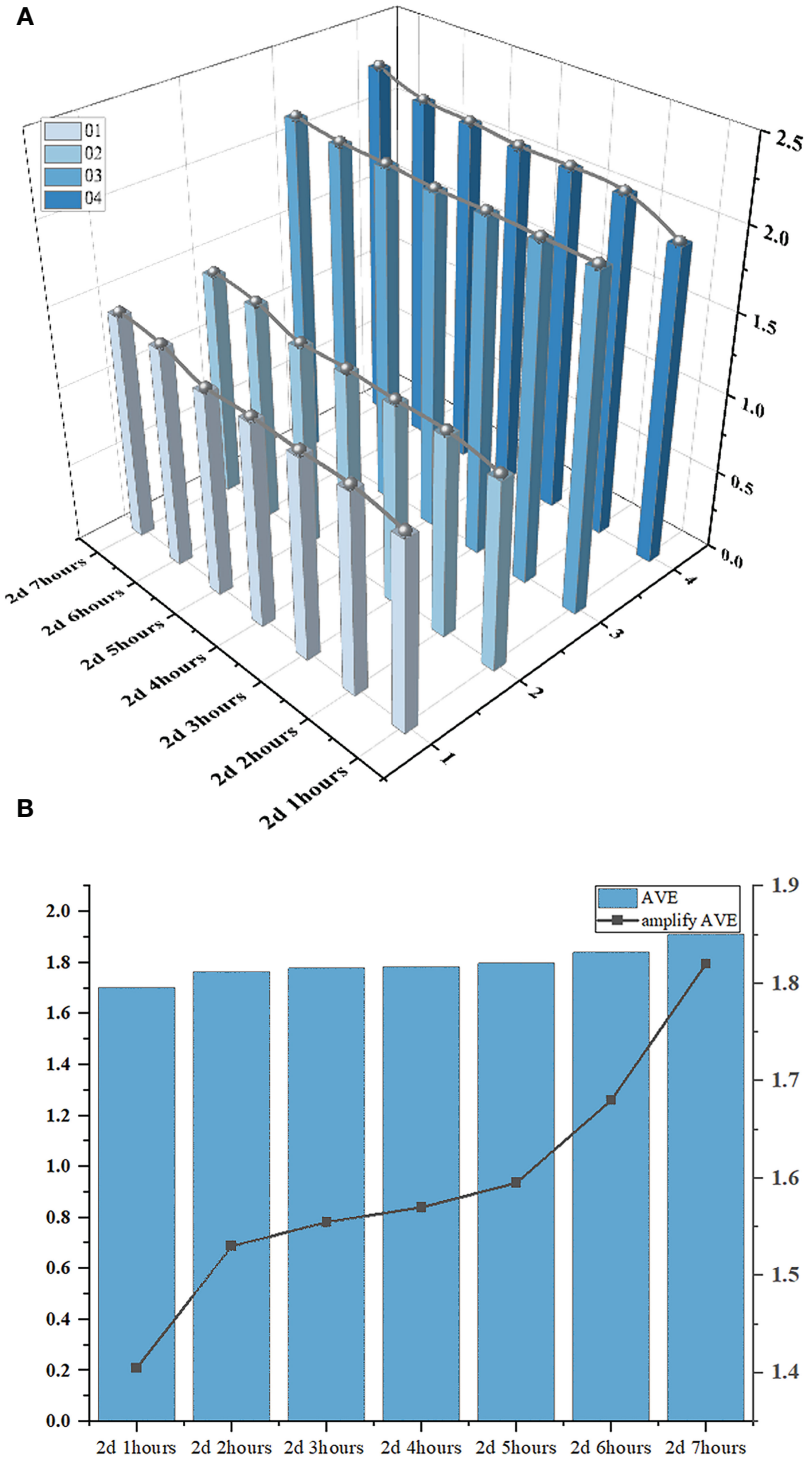
**(A)** is 3D bar chart of the changes in the radicle length of soybean seeds over time. **(B)** is the bar chart of the average radicle length and the line graph of the amplify of the ‘AVE’.

To explore the patterns of soybean radicle feature changes, based on the conclusions drawn from the radicle lengths of 1, 2, 3, 4, we have plotted a 3D bar chart (**Figure 14A**) illustrating the changes in radicle length of the soybean seed over time. In order to magnify the trend of radicle length changes for easier observation and to reduce the systematic errors generated during machine recognition of radicle lengths, we subtracted 1 from the average radicle length of the four soybean seeds during the same time period and then multiplied the result by a scaling factor of 2 to represent the growth curve (depicted as the “amplify AVE” line segment in **Figure 14B**). It is easy to determine the growth rate of the soybean radicle during this specific period from the slope of the line in the line chart. From 2d 1hour to 2d 2hours, the radicle growth rate was high. From 2d 2hours to 2d 5hours, the line chart remained stable, indicating no significant changes in the radicle lengths of the four seeds. However, from 2d 5hours to 2d 7hours, the slope of the line chart was the greatest, indicating that the radicle growth rate reached its maximum during this time period, and it continued to maintain a relatively fast growth rate thereafter.

**Figure 15:**
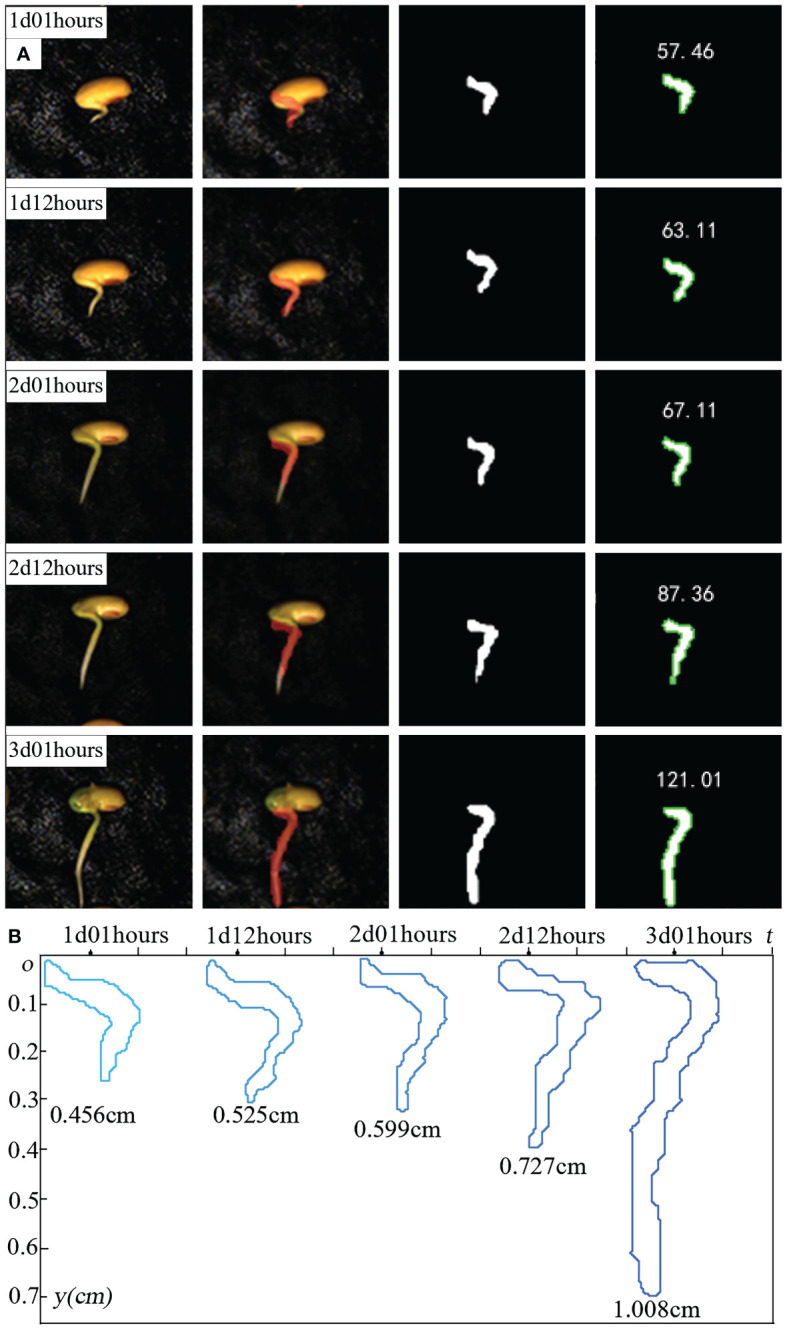
**(A)** monitoring of radicle characteristics of individual soybean seeds throughout the entire experimental cycle. **(B)** Overlay each contour onto the coordinate axis.

We also utilized our system and model to complete the segmentation monitoring of the radicle characteristics of a selected soybean seed over a longer experimental period. The images selected in **Figure 15A** were taken at 12-hour intervals, and the segmented calculation yielded the number of pixels in the radicle frame. From the figure, it is evident that the growth trend of the soybean seed throughout the germination period can be analyzed. From 1d 1hours to the 2d 12 hours, the radicle of the soybean seed exhibited slow growth, with the number of contour pixels increasing at a relatively steady pace. From the 2d 12 hours to the 3d 1 hours, the radicle of the soybean seed developed rapidly, and the contour noticeably expanded. To better illustrate the changes in the radicle contour of the soybean throughout the entire experimental period, we extracted the contours from (**Figure 15A**), magnified them proportionally, and superimposed them onto the coordinate axis (**Figure 15B**). The unit length of this coordinate axis is 0.1 cm, and the numbers below the contours represent the actual length of the radicle. This method can better assist us in assessing the radicle development of the selected seed and quantitatively analyzing the changes in radicle characteristics.

The method described above demonstrates that our system successfully completed the full-time sequence detection of soybean radicle characteristics, generating profiles that change over time. This provides data support for further research into the regularity and trends of radicle feature changes during soybean growth. We anticipate that through this continuous analysis, we will gain a deeper understanding of the characteristics of soybean radicles at different growth stages, providing reference and guidance for crop growth regulation and optimization.

Researchers commonly use YOLO models for crop root classification and identification. For instance, Li et al. (2022) utilized YOLOv4 to identify root objects in ground-penetrating radar images, while Yang et al. (2022) enhanced YOLOv2 for garlic root recognition and automatic slicing. In comparison to these studies, our model employs image segmentation for embryo radicle extraction, enabling high precision and pixel-level embryo radicle feature extraction. The improved segmentation module directly outputs binary images with radicle masks, addressing issues of redundant information in traditional segmentation modules that generate color-rich and information-dense original images. This allows the subsequent image processing to focus more effectively on soybean radicle features. The integrated computational module in our model enhances its functionality by calculating the segmented soybean radicle length. In contrast to the method of quantifying coarse roots using ground-penetrating radar (Guo et al., 2013), our model can detect, segment, and quantify fine roots, filling a gap in root system analysis methods for traits with small characteristics. Compared to directly measuring root length using image analysis (Kimura et al., 1999), our approach segments the radicle portion in images before measuring root length, simplifying the calculation process and reducing the required image sample size.

The continuous time-series tracking of soybean radicle is achieved through a combination of software and hardware. On the hardware side, utilizing the apparatus we have constructed allows for high-definition, long-term, continuous imaging of soybean radicles. The captured images are then sorted chronologically and input into our model to generate images with specific root lengths. By arranging these images in sequence, one can discern the temporal changes in seed development. Analyzing the complete temporal characteristics of seeds using traditional methods entails significant labor costs and is susceptible to interference from variations in factors such as light and temperature that affect radicle traits (Gibson and Mullen, 1996). However, the collaboration between our equipment and model enables robust, cost-effective, and precise temporal tracking of soybean radicle features.

## 4 Summary, limitations and future work

To address the time-consuming, subjective, inefficient, and inaccurate nature of traditional soybean seed radicle feature research, and taking into account the detection challenges posed by the intertwining and overlapping of radicles during soybean germination, this paper proposes a soybean seed radicle feature segmentation and length calculation model: YOLOv8-segANDcal. Firstly, we augmented the original YOLOv8 backbone structure with a novel convolutional attention framework, SegNext_Attention, to assist the model in better detecting and segmenting soybean radicles, thereby enhancing its focus on the roots of soybeans and improving detection accuracy. Secondly, we improved the original segmentation head to output both the segmented mask information image and the corresponding binary image, enhancing the practicality of the model’s output and facilitating subsequent image processing. Additionally, we incorporated the Cal module into YOLOv8-segANDcal, endowing the model with the capability to calculate the number of border pixels in the binary image. Finally, we presented a method for calculating radicle length, obtaining the actual length of soybean radicles and capturing the changes in radicle contour features over germination time, thereby contributing to the study of soybean radicle characteristics.

The experimental results show that YOLOv8-segANDcal achieved a segmentation mAP_50-95_ of 36.8% and a detection mAP_50-95_ of 72.2%, representing the highest accuracy in soybean radicle segmentation. Compared to the unimproved YOLOv8-seg, there was an improvement of 2% and 1% respectively. The improved Segment module and the added Cal module enabled the model to output more information and calculate the average length of the selected soybean radicles as 2.71cm, with a median length of 2.50cm. The linear correlation of 0.9737 between manual and machine vision measurements demonstrates the feasibility of the YOLOv8-segANDcal model in practical soybean radicle length calculations. The extraction of radicle contour features can aid in studying the morphological evolution of radicles over germination time, facilitating the assessment of soybean seed vitality. This is the first application of the YOLOv8-seg model in the analysis of soybean seed radicle features, and we have developed a rapid and accurate method for extracting and calculating soybean seed radicle features. However, this method still has limitations. For example, the model size is larger than YOLOv8, increasing deployment difficulty. Additionally, there is the potential for misidentification during radicle segmentation, particularly in the early stages of soybean germination, where short radicles may be mistaken for background noise, leading to false detections.

In the future, we will continue to develop our model, utilizing lightweight methods to reduce model size while maintaining segmentation accuracy. We plan to integrate sensors for pressure, humidity, and other environmental factors into our experiments, feeding sensor data into the model to obtain additional soybean seed radicle characteristics such as radicle fresh weight and dry weight. Our YOLOv8-segANDcal model has achieved significant results in the feature extraction of soybean radicles, which inspires us to explore its potential applications in other crops and various growing conditions. For instance, we could apply this technology to rice, especially during its seedling stage, to segment and calculate leaf area and the length of roots to assess their efficiency in absorbing water and nutrients. Another example is wheat planted under drought conditions, where our model can help monitor root system development to evaluate its drought resistance. Moreover, our model could be applied to tomatoes grown in greenhouse environments, analyzing the morphological characteristics of fruits and leaves to optimize irrigation and fertilization schedules. For crops like cotton grown in saline-alkali soils, the model can be used to monitor and assess the crop’s salt tolerance by measuring changes in leaf morphology and root features.

We believe that with further research and model adjustments, YOLOv8-segANDcal can become a versatile tool in the field of smart agriculture, not only for monitoring crop growth but also for predicting crop diseases and pest infestations, thereby increasing crop yield and quality, assisting crop breeders and agronomists in rapid breeding and variety selection.

## Data availability statement

The raw data supporting the conclusions of this article will be made available by the authors, without undue reservation.

## Author contributions

YW: Conceptualization, Data curation, Formal analysis, Funding acquisition, Investigation, Methodology, Project administration, Resources, Software, Supervision, Validation, Visualization, Writing – original draft, Writing – review & editing. ZL: Formal analysis, Investigation, Resources, Software, Supervision, Validation, Writing – original draft, Writing – review & editing. HJ: Data curation, Investigation, Software, Visualization, Writing – review & editing. QL: Methodology, Project administration, Supervision, Writing – review & editing. JQ: Investigation, Resources, Writing – review & editing. FP: Resources, Visualization, Writing – review & editing. XF: Formal analysis, Project administration, Supervision, Writing – review & editing. BG: Funding acquisition, Project administration, Supervision, Writing – review & editing.
